# Discovery of biomarkers in rare diseases: innovative approaches by predictive and personalized medicine

**DOI:** 10.1186/s13167-016-0074-2

**Published:** 2016-12-08

**Authors:** Basri Gülbakan, Rıza Köksal Özgül, Ayşe Yüzbaşıoğlu, Matthias Kohl, Hans-Peter Deigner, Meral Özgüç

**Affiliations:** 1 Pediatric Metabolism Unit, Institute of Child Health, Hacettepe University, Ankara, Turkey; 2 Department of Medical Biology & Biobank for Rare Disease, Faculty of Medicine, Hacettepe University, Ankara, Turkey; 3 Institute of Precision Medicine, Medical and Life Sciences Faculty, Furtwangen University, Villingen-Schwenningen, Germany; 4 Fraunhofer Institute IZI, EXIM Department, Rostock, Germany

**Keywords:** Rare diseases, Biomarker panels, Multi-omics, Predictive preventive personalized medicine, Innovation, Prognosis, Screening, Individualized therapy, Biobank, Drug targets

## Abstract

There are more than 8000 rare diseases (RDs) that affect >5 % of the world’s population. Many of the RDs have no effective treatment and lack of knowledge creates delayed diagnosis making management difficult. The emerging concept of the personalized medicine allows for early screening, diagnosis, and individualized treatment of human diseases. In this context, the discovery of biomarkers in RDs will be of prime importance to enable timely prevention and effective treatment. Since 80 % of RDs are of genetic origin, identification of new genes and causative mutations become valuable biomarkers. Furthermore, dynamic markers such as expressed genes, metabolites, and proteins are also very important to follow prognosis and response the therapy. Recent advances in omics technologies and their use in combination can define pathophysiological pathways that can be drug targets. Biomarker discovery and their use in diagnosis in RDs is a major pillar in RD research.

## Background

Diseases defined as “rare” have a very low prevalence; with EU definition of 1 patient per 2000 individuals. In EU, it is estimated that about 6–8 % of the population is affected with rare diseases (RDs) which makes about 30 million individuals. Worldwide, there are about 350–400 million rare disease patients (https://globalgenes.org/rare-diseases-facts-statistics).

Even if a single RD has a low prevalence, there are more than 8000 different diseases which make them a formidable health problem. About 80 % of RDs have a genetic origin and affect pediatric age group even though there are diseases that manifest at later ages. Most RDs are very severe, chronic, and life threatening and have not yet been well characterized. There is a general lack of knowledge which makes diagnosis difficult and most of the patients receive a very delayed diagnosis after consulting with multiple healthcare centers. Due to low numbers, clinical trials are challenging and development of drugs has been hampered since these diseases have not caught the attention of large pharmaceutical companies.

In recent years, there are national and international initiatives to accelerate research for timely diagnosis and development of new therapies for RDs [1] (http://www.ema.europa.eu/ema/index.jsp?curl=pages/special_topics/general/general_content_000034.jsp).

## European activities for rare diseases

In 2008, European Commission has published a communication (COM 679/2) indicating challenges of RDs and sets a strategy for increasing the visibility of and the cooperation and coordination for RDs in Europe. This recommendation has initiated the formulation of national plans for rare diseases in the member states. Furthermore, regulatory bodies such as European Medicinal Agency (EMA) [2] in EU and EC Orphan Drugs Regulation that European Parliament and the Council adopted [(CE) No. 141/2000, (CE) No. 847/2000] and 1983 Orphan Drugs Act in the USA [3] are supporting the development of orphan drugs through different incentives for the pharmaceutical companies.

## International activities for rare diseases

A most recent development is the initiative “The International Rare Diseases Consortium (IRDiRC)” “by the collaborative action of USA, Canada, and EU”, IRDiRC aims to foster collaborative research efforts and harmonize policy for accelerating diagnosis and therapy of rare diseases. The consortium has set the following general goals: “establishing and providing access to harmonized data and samples, performing the molecular and clinical characterization of rare diseases, boosting translational, preclinical and clinical research, streamlining ethical and regulatory procedures.” without the mission of finding 200 new therapies and diagnosis of all RDs by the year 2020 [4].

Altogether, the roadmap for a concerted action for RDs needs infrastructures for well-characterized and organized collections of biological samples (biobanks) for biomarker discovery, patient registries with well-defined phenotypes that are linked to the biological samples and omics platforms (genomics, transcriptomics, proteomics, metabolomics) that will help to uncover the pathophysiology of still uncharacterized diseases. In vitro and in vivo models will aid to annotate the function of the newly discovered genes and the development of new modalities of therapies. Moreover, bioinformatics tools are essential to harmonize the high-throughput data generated by the omics platforms.

The impact of personalized medicine approach on health care is being felt already in clinical practice. The use of integrated omics technologies is the driving force in personalized medicine for biomarker discovery. The development of genetic tests based on biomarker discovery will be basic accelerators for better diagnosis and targeted therapies in RDs [5–7].

## Types of biomarkers

A biomarker in general denotes characteristics assigned to a biological state and/or change. While this may include physical methods, such as EEG or ECG, biomarker in a more confined biomedical context comprise biological entity(ies) that can be used for the diagnosis, prognosis of disease, and individual’s response to drugs or therapies (a characteristic that is objectively measured and evaluated as an indicator of normal biologic processes, pathogenic processes, or pharmacologic responses to a therapeutic intervention) [8]. The following parameters are indicators of a biomarker that is reliable and is valuable in the clinical setting.

1. A biomarker needs to have a clinical and analytical validity.
2. It should be measured by tests that are reliable, accurate, and reproducible and should distinguish between the pathological and healthy state.
3. The biomarker should also be able to indicate any changes in the status of the disease and the disease development in a stable manner and not be influenced by outside parameters. A clinically validated biomarker should indicate the prognosis of a disease or an individual’s response to a drug.

In rare diseases that have a genetic origin, causative genes, disease-causing mutations, polymorphisms, and phenotypic dynamic markers, i.e., RNA/miRNAs, proteins, and metabolites that can change over time are all considered valuable biomarkers to identify/characterize the disease as well as the cellular pathophysiology. For clinical utility, biomarkers should be measured in biological samples obtained by non-invasive methods such as urine, stool, plasma, serum, and saliva rather than biopsies. Ideally, the biomarker activity needs to remain stable in the biological sample used [9, 10].

The major goals in the RDs research are molecular classification, identification of new genes, and determination of causative mutations, biomarker discovery, development of new diagnostics and therapies, and establishment of high-quality sample biobanks and patient registries. It goes without saying that a biomarker for a complex disease nowadays usually comprises several characteristics, such as a combination of metabolites, transcripts, or peptides.

## Genomics and transcriptomics

Initial gene identification studies relied on the large families with multiple affected individuals. Using this approach, mapping a genomic interval means that many genes in this region need to be sequenced to find the mutation responsible for the disease that segregates in the family. The positional cloning approach relied on the genomewide use of microsatellite markers or SNPs. In populations where inbreeding ratio is high, homozygosity mapping is a valid approach for identification of genes in autosomal recessive (AR) monogenic diseases [11].

The latest advances in genetic technologies are facilitating the use of whole-exome (WES) or whole-genome sequencing (WGS) for identification of disease genes [12]. Coupled with advanced bioinformatics techniques, this approach is being used successfully in RDs research and is also entering the clinic for diagnostic purposes. The advantage of WES approach is that it is faster and cheaper than WGS. The exonic sequences that make up about 1 % of the genome are reported to harbor more than 80 % of the disease-causing mutations [13]. The advantage of the WGS approach is that by this technique, all exons and non-coding genomic sequences are covered and structural variations can also be detected. The identification of non-coding genomic variations that can act as modifier can be informative for explanation of discordant spectrum of phenotypes that is frequently observed in rare diseases [14].

Cellular gene expression patterns are known to change in health and disease. Global transcriptome assays now can be done using the RNA-Seq technology that utilizes the next generation sequencing methodology. Besides detecting cDNA sequences, the method is also capable of detecting miRNAs. miRNAs are non-coding short (20–22 nucleotides) RNA molecules that are involved in the regulation of cellular pathways. In this respect, they have emerged as valuable diagnostic biomarkers and they can also be used as markers with respect to the response to therapy [15]. A recent study has shown that circulating miRNAs are found to be elevated in serum of patients with Duchenne and Becker muscular dystrophies. The level of the identified miRNAs decreased after exon skipping therapy and restoration of dystrophin protein [16].

Another rare disease where alterations in miRNAs have been observed is Rett syndrome, a severe neurological disorder. It has been observed that significant alteration of miRNA expression patterns occurs in mice with disease-causing mutations in the Mecp2 protein [17]. As next generation sequencing technologies (NGS) are more commonly used, it is expected that the identification of miRNAs as circulating biomarkers will facilitate the therapeutic and prognostic testing in rare diseases.

## Metabolomics

Rare diseases are a heterogeneous group of diseases with small number of patients that need better analytical tools and approaches for diagnosis and treatment. Unfortunately, many undiagnosed cases are fatal, and a large group of these patients are affected with neurometabolic symptoms. Metabolomics as an approach has the advantage to detect alterations and deficiencies in the metabolic state that is a cellular marker for as a molecular signature. Since body fluids can be used for metabolomics, it is a non-invasive approach that can lead to new diagnosis and grading of the disease. Also, the identification of the affected biochemical pathways can act as targets for drug discovery which is still in the management of rare diseases.

Metabolomics is defined as the global systematic study of the unique chemical fingerprint end products of the metabolic transformations that occur in the biological systems [18, 19]. As the newest member of the omics family, metabolome refers to the low-molecular weight (<1500 Da) molecules such as amino acids, lipids, carbohydrates, biogenic amines, and organic acids that biochemical reactions leave behind [20]. Cellular pathways can be followed on a real time basis by metabolomic studies, and it better reflects the actual metabolic state of a cell, tissue or organ, translating the genotype and environmental factors into the phenotype. Therefore, metabolites usually serve as a clinical endpoint and delineate the disease mechanisms and reflect the underlying biochemistry [21]. It is for this reason that many metabolites are also used as biomarkers for different diseases. Metabolome consists of different molecule classes that have very different chemical structures. Accordingly, no single analytical tool is able to cope with the huge chemical diversity of the metabolome. Multiple analytical tools are needed to get a comprehensive and complete picture of the whole metabolome. Although many different analytical tools are used in metabolomics, nuclear magnetic resonance (NMR) [22] and mass spectrometry (MS) [23] stand out as the most powerful and information-rich techniques. In NMR, samples are directly analyzed with minimal sample preparation. This technique eliminates the time-consuming separation and derivatization steps and is also a non-destructive technique. Moreover, the features of different metabolites are quite distinguishable especially when high magnets are used. However, the relatively poor sensitivity of NMR (mM to high uM), requirement of high sample volumes (high uL to ml range), and very high instrument costs are still the main drawbacks of this technique. The other primary tool used in metabolomics is gas or liquid chromatography equipped with mass spectrometry (GC-MS and LC-MS). Both GC-MS and LC-MS are quite sensitive (uM to nM range) and amenable to automation. On contrary to NMR spectroscopy, very low sample volumes (uL) are required in mass spectrometric methods. This technique became particularly useful in studies involving human subjects where biological samples amount is an issue. Today, MS has therefore become the method of choice for many metabolomics laboratories worldwide. In some recent studies, NMR spectroscopy is used orthogonally to mass spectrometry techniques to improve the metabolome coverage and get a more detailed picture [24].

## Metabolomics methods

Metabolomics studies are usually condensed into two main categories as targeted and untargeted metabolomics. In *untargeted* metabolomics, the question of “which metabolite(s) are different in particular sets of samples” is answered. Untargeted metabolomics provides an unbiased and global profile of the samples, and these profiles are used to extract statistically different metabolites between control and test samples. Following stringent bioinformatic analyses and database search, the evolved data is linked to biochemical pathways, and therefore this approach is also known as “hypothesis generating metabolomics”. Upon determining statistically significant metabolites, they are then subjected to validation. Untargeted global profiling is also named as “next generation metabolomics” akin to “next generation sequencing” [25].

In *targeted* metabolomics, as its name implies, certain metabolites are predefined and these metabolites are probed across different samples to determine their fold changes. The question of “if the quantity of a certain metabolite(s) is different in two different states” is answered. Unlike untargeted metabolomics, prior biochemical knowledge is used to define the metabolites to be quantified. This method is used complementary to the untargeted metabolomics to validate putative biomarkers. As it is biased towards the metabolites of choice, it is very selective, much faster and greater sensitivities can be attained [26].

## Metabolomics in rare disease diagnosis and characterization

While metabolomics is a fairly new omics area, the idea of using metabolites for screening rare diseases is indeed quite established. In 1960s, Robert Guthrie introduced the bacterial inhibition assay, and this assay is still being used as a semiquantitative technique in early screening of phenylketonuria. After the development of triple quadrupole mass spectrometers, such kind of assays were replaced by mass spectrometry methods. This technique which is commonly known as “tandem MS” in clinical lab practice essentially relies on the “targeted metabolomics” principles. If disease-specific metabolites are known, then MS assays for these “targets” can be very specifically developed [27, 28].

The main advantage of this approach is that many diseases can be screened in a single experimental run simultaneously. For the past two decades, tandem mass spectrometry has been used for screening well over 60 different diseases and identified clinical biomarkers can be used to explain the pathological phenotypes. Rare diseases including inborn errors of metabolism are the prime examples of those groups of diseases.

Organic acidemias, amino acid disorders, fatty acid oxidation defects, congenital disorders of glycosylation, and lysosomal storage diseases can now be routinely screened by specific MS methods. Henceforth, MS became the centerpiece instrumentation of clinical laboratories. A list of some rare metabolic diseases that could be diagnosed by targeted metabolomics is illustrated in Table 1 [29].

**Table 1:** Disease-specific analytes by targeted metabolomics

Diseases	Disease-specific analytes
PKU	Phe
MSUD	[Leu + Ile] and Val, alloisoleucine
Homocystinuria	Met
Citrullinemia I, argininosuccinic aciduria, citrin deficiency	Cit
OTC deficiency, CPS1 deficiency	Cit (low)
Arginase deficiency	Arg
Tyrosinemia	Tyr
Systemic carnitine deficiency	C0 (low)
CPT1 deficiency	C0 (high)
Methylmalonic aciduria, propionic aciduria, cobalomanine defects	C3
SCAD deficiency, IBD deficiency	C4
SCHAD (HAD) deficiency, ketosis	C4-OH
Isovaleric acidemia	C5
Glutaric aciduria type 1	C5-DC
Glutaric aciduria type 2	C4-C18
Multiple carboxylase deficiency, 3-methylcrotonylglycinuria, 3-methylglutaconicaciduria I, 3-oxothiolase, HMG uria, beta-ketothiolase deficiency, biotinidase deficiency	C5-OH
MCAD deficiency	C6, C8-C10-C10:1
VLCAD deficiency, ketosis	C12, C12:1, C14, C14:1, C14:2, C16, C18, C18:1
CPT2 deficiency	C16, C18:1, C18:2
TFP	C14, C14:1, C14:2, C16-OH, C16:1-OH, C18:1, C18:2
LCHAD	C14, C14:1, C14:2, C16-OH, C16:1-OH, C18:1, C18:2
CACT deficiency	C18:1(C18)

## Data analysis

The analysis of all kind of omics data consists of similar steps. It starts with a thorough quality control of the raw data. Next, one or more preprocessing steps are applied to the raw data. This is also called low-level analysis and for instance can include background/baseline correction and normalization. The goal of preprocessing is to account for the various sources of variation which are immanent in omics experiments. It is necessary as many statistical procedures rely on homoscedasticity or distributional assumptions which are surely not fulfilled for the raw data [30]. Then, the actual statistical analysis is conducted using the preprocessed data. Here, all kind of uni- and multivariate statistical methods are applied dependent on the question at hand. There are associated statistical method dependent recent developments in genomics [31]. The analysis of high-dimensional omics data, however, can be quite challenging especially because of the “large p, small n” problem; that is, the number of features/variables (e.g., genes, metabolites, etc.) in such omics data sets is as a rule (much) larger than the number of observations [32]. There are statistical challenges of high-dimensional data [33]. Finally, information from various databases is added to the features obtained by the statistical analysis. First, one assigns various categories (provided by the databases) to the features where the categories are based on, e.g., functional, biological, chemical, health, etc. information [34, 35]. Then, by applying the so-called enrichment methods, one identifies the categories that are over-represented; that is, include more features than one would expect by chance [36, 37]. These over-represented categories represent specific properties of the features identified in the statistical analysis and enable a deeper understanding and further interpretation of the results.

## Biobanks for rare diseases

Omics technologies require the use of well-annotated biological samples of high quality. Biobanks that meet the quality assurance criteria and with sound ethical and legal guidelines are valuable infrastructures that house biological samples as well as the clinical data associated with the samples. Recent developments have led to networking activities in biobanks across Europe. EU has supported the formation of European Research Infrastructure Consortiums (ERICs) [38] and Biobanking and BioMolecular Resources Research Infrastructure (BBMRI-ERIC) [39] is a pan-European platform of member and observer states towards creation of and sharing of the best practice guidelines as well as harmonized ethical legal and social issues (ELSI) that will enable sharing of samples and data across the EU borders. BBMRI gained legal status in 2013 and main advantage of ERICs as governance structures will be a long-term cooperation among states that will enable sustainability which is crucial in biobanking activities.

In rare diseases, a well-established network in biobanking activity is EuroBioBank. Twenty one biobanks from nine European countries are currently members of The EuroBioBank. The primary goals of the network are as follows “Identify and localise biological material of interest to researchers, build a critical mass of rare disease sample collections, distribute high quality material and associated data to users, promote best-practice guidelines for biobanking activities, disseminate knowledge and know-how to the scientific community through training courses, enhance collaboration with the medical and scientific community in the field of rare diseases” [40].

Biobanks have become a crucial step in the translational process of the PPPM [41–43]. Since the number of rare disease patients in each country is rather small, especially for clinical trials and development of new treatments, the use of biobank samples within networks will facilitate better management of rare disease patients [44]. To be able to use biomarkers as diagnostic tools in personalized medicine, there are challenges such as high cost of technology, management of high-throughput data and education of healthcare professionals for interpretation of omics data. Furthermore, ethical and legal guidelines supporting the use of genomics technologies will highly facilitate the personalized medicine approach in RDs.

## Conclusions

Personalized medicine has a high potential in translating research results to clinical practice. Given the fact that undiagnosed diseases still constitute the largest portion of the rare diseases cohorts, advances in the high-throughput omics technologies will enable identification of better diagnostic and prognostic biomarkers. Characterizing diseases at the molecular level helps to unravel biochemical pathways that are potential drug targets. However, the basic intrinsic problem is that the analysis of large cohort of rare disease samples is quite difficult as there are limited reported patients. This difficulty could only be overcome by national and international collaborative efforts and multicenter data sharing. Education of healthcare personnel, policymakers, and awareness raising in the general public is needed to realize the goals of personalized medicine.

In summary, in the next 20 years, personalized medicine will be at the forefront of clinical applications, and customizing the individualized treatment will be guided by the individual’s specific biomarker panels. Especially in rare diseases where early diagnosis and treatment is still a bottleneck, a personalized medicine approach will have a high impact on increasing the quality of medical care of rare disease patients.
